# Evaluation on Elongation Factor 1 Alpha of *Entamoeba histolytica* Interaction with the Intermediate Subunit of the Gal/GalNAc Lectin and Actin in Phagocytosis

**DOI:** 10.3390/pathogens9090702

**Published:** 2020-08-27

**Authors:** Hang Zhou, Yue Guan, Meng Feng, Yongfeng Fu, Hiroshi Tachibana, Xunjia Cheng

**Affiliations:** 1 Department of Medical Microbiology and Parasitology, School of Basic Medical Sciences, Fudan University, Shanghai 200032, China; 18111010069@fudan.edu.cn (H.Z.); 13111010025@fudan.edu.cn (Y.G.); mengfeng@fudan.edu.cn (M.F.); yffu@fudan.edu.cn (Y.F.); 2 Department of Infectious Diseases, Tokai University School of Medicine, Kanagawa 259-1193, Japan; htachiba@is.icc.u-tokai.ac.jp

**Keywords:** *Entamoeba histolytica*, elongation factor 1 alpha, Gal/GalNAc lectin, actin

## Abstract

*Entamoeba histolytica* is the causative agent of amoebiasis. This disease results in 40,000 to 100,000 deaths annually. The pathogenic molecules involved in the invasion of trophozoites had been constantly being clarified. This study explored the role of elongation factor 1 alpha (EF1a) in *E. histolytica* pathogenicity. Biolayer interferometry binding and pull-down assays suggest that EF1a and intermediate subunit of lectin (Igl) binding are specific. Submembranous distribution of EF1a closely aligns with the localization of Igl, which appear in abundance on membranes of trophozoites. Messenger RNA (mRNA) expression of EF1a is positively correlated with trends in Igl levels after co-incubation with Chinese hamster ovary (CHO) cells in vitro, suggesting a regulatory linkage between these proteins. Erythrophagocytosis assays also imply a role for EF1a in phagocytosis. Finally, EF1a and actin are collocated in trophozoites. These results indicated elongation factor 1a is associated with *E. histolytica* phagocytosis, and the relationships between EF1a, Igl, and actin are worth further study to better understand the pathogenic process.

## 1. Introduction

*Entamoeba histolytica* (*E. histolytica*) causes amoebiasis, a serious public health problem in both developing and developed countries. Amoebiasis causes an estimated 50 million cases of dysentery, colitis, and extraintestinal abscesses, resulting in 40,000 to 100,000 deaths annually [1]. Galactose (Gal)- and N-acetyl-D-galactosamine (GalNAc)-inhibitable lectin plays a significant role in the development of amoebiasis by supporting adherence of *E. histolytica* trophozoites to colonic mucins and host cells [2]. Monoclonal antibodies (mAbs) toward the intermediate subunit of lectin (Igl) block trophozoite adherence to mammalian cells in vitro [3,4,5]. Igl are thus involved in *E. histolytica* pathogenicity, but molecules involving pathogenicity that work in concert with Igl remain unclear.

Elongation factor 1 alpha (EF1a) captured our attention for its possible interaction with the C-terminal fragment of Igl (Igl-C). EF1a is an essential and highly conserved protein ubiquitously expressed in eukaryotic cells. It is known that EF1a is an essential “housekeeping” protein that effected on the guanosine triphosphate (GTP)-dependent binding of aminoacyl-tRNA to the A-site of ribosomes during protein synthesis, and EF1a serves as an essential hub in protein networks [6]. In addition, the factor may also be involved in other cellular processes [7,8,9], including apoptosis, signal transduction, tumorigenesis, and cytoskeletal regulation.

There are an interesting fact that the 12 amino acid deletions in EF1a have been found in some pathogenic protozoans of major global health importance, including *Leishmania donovani* [10,11], *Plasmodia* spp. [12], *Giardia lamblia* [13], *Toxoplasma gondii* [14], *Cryptosporidium parvum* [15,16], *Trypanosoma cruzi* [17], *T. brucei brucei*, and *Entamoeba histolytica* [10,18]. The same amino acid sequence deletion compared with human EF1a, indicated targeting the exposed region of EF1a common to all of these pathogens could have far-reaching benefits.

In the present study, we investigated molecular cloning and expression of EF1a and Igl in the trophozoites, analyzed cellular localization of these proteins, and explored erythrocyte phagocytosis. Several approaches are currently in progress, attempting to develop pathogenic molecules related to pathogenesis. We have described a novel protein co-locations relationship between Igl, actin, and EF1a of *E. histolytica*. We report the successful results of this endeavor, which show that EF1a could specifically bind to pathogenic lectin Igl and co-localization with actin. Targeting of EF1a might prove to be efficacious consider that it is both essential for *E. histolytica* survival and known to be involved in pathogenesis. Thus, providing a basis strategy for novel drug target development for treatment amoebiasis.

## 2. Results

### 2.1. Specific Binding of EF1a to Igl-C

**Figure 1:**
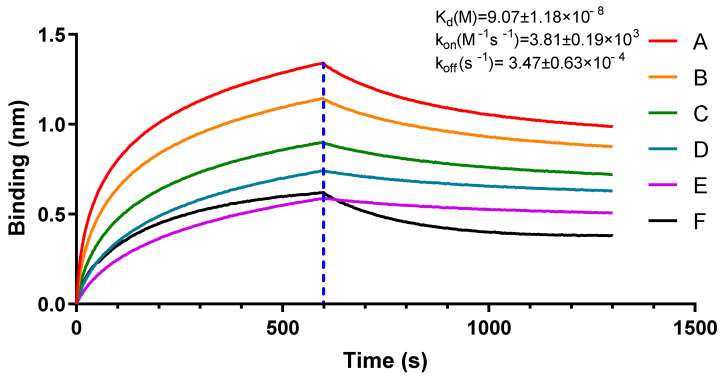
Binding affinity of rEF1a to Igl-C and BSA measured by Octet-RED 96 (Pall ForteBio). Purified Igl-C (biosensors A–E) and BSA (biosensor F) were immobilized on activated AR2G biosensors. Analytes for biosensors A–E were serial dilutions of rEF1a proteins from 4000 to 250 nM. Analytes for biosensors F rEF1a proteins were at 4000 nM. Binding kinetics were evaluated using the 1:1 Langmuir binding model in Fortebio Data Analysis 8.0 Software.

Protein mass spectrum showed high UniquePepCount of protein EF1a (No.3–No.7) indicating an interaction between protein Igl-C and EF1a. (Supplementary Table S1) Then, we measured the binding kinetics of the recombinant protein rEF1a with Igl-C using biolayer interferometry with an Octet-RED system (Pall Forte Bio). Igl-C exhibited a high binding affinity for rEF1a (Figure 1). The equilibrium dissociation constant (KD) of rEF1a for Igl-C was (9.07 ± 1.18) × 10^−8^ M with an on-rate (k_on_) of (3.81 ± 0.19) × 10^3^ M^−1^s^−1^ and off-rate (k_off_) of (3.47 ± 0.63) × 10^−4^ s^−1^. Immobilized bovine serum albumin (BSA) showed little binding with rEF1a (Figure 1) with a much lower association rate and higher dissociation rate, demonstrating specific rEF1a and Igl-C binding.

### 2.2. Antibody Specificity Confirmed by Western Blotting

**Figure 2:**
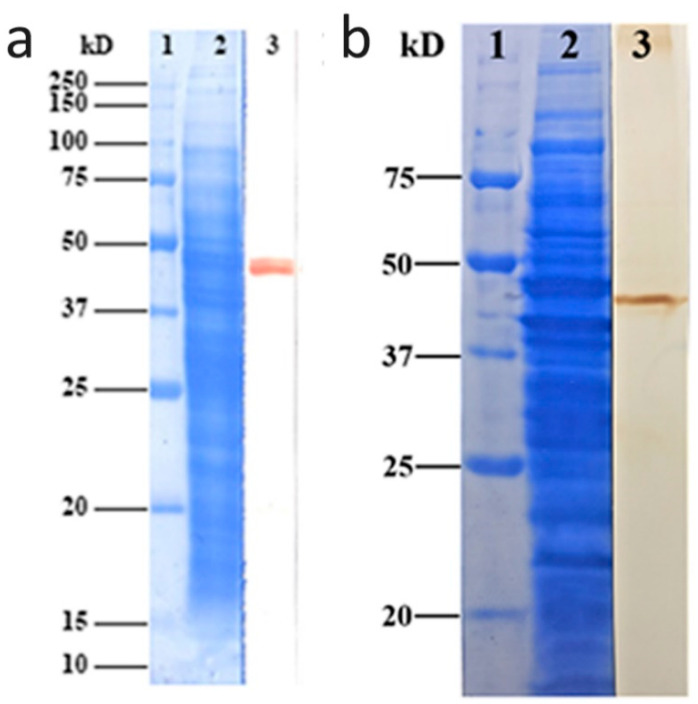
(**a**) Immunoblotting of native *E. histolytica* EF1a to test the specificity of the rabbit anti-eEF1a MAb. Lane 1: standard protein molecular weight marker; Lane 2: trophozoite lysates; Lane 3: native EF1a probed by the rabbit anti-eEF1a MAb from trophozoite lysates. (**b**) Immunoblot for native E. histolytica EF1a to test specificity of polyclonal antibodies. Lane 1: standard protein molecular weight marker; Lane 2: trophozoite lysates; Lane 3: native EF1a probed by the polyclonal antibodies against rEF1a (dilution of 1:4000) from trophozoite lysates.

We verified the specificity of the rabbit anti-eEF1a MAb (catalog no. ab157455; Abcam, Cambridge, UK) with Western immunoblotting. A single immunoband was observed on the polyvinylidene fluoride (PVDF) film at about 48 kDa (Figure 2a). Polyclonal antibodies against rEF1a were used to detect native EF1a protein in *E. histolytica* lysates. The 48 kDa band displayed antibody binding, demonstrating the reliability of polyclonal antibodies for subsequent indirect immunofluorescence and erythrophagocytosis assays (Figure 2b).

### 2.3. Localization of Igl and EF1a

**Figure 3:**
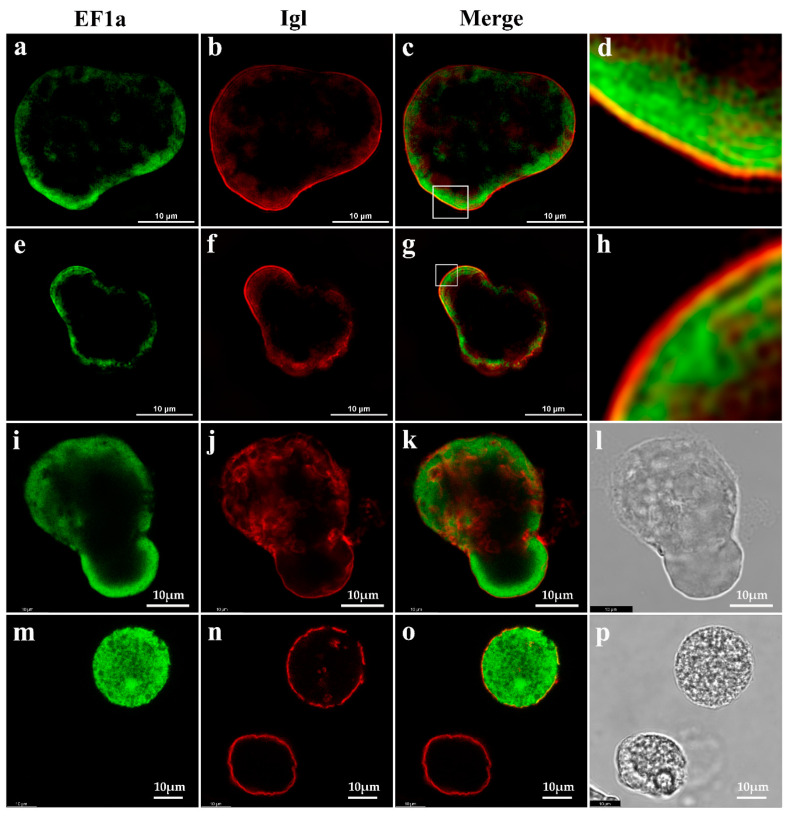
Localizations of EF1a and Igl in trophozoites. Part 1. Fluorescence staining of fixed cells observed by N-SIM (**a**–**h**) and confocal microscopy (**i**–**l**). (**a**,**e**,**i**) staining with rabbit anti-eEF1a MAb; (**b**,**f**,**j**) staining with monoclonal antibody EH3015 for Igl; (**c**,**g**,**k**) merger of (**a**,**e**,**i**) and (**b**,**f**,**j**); (**d**,**h**) fractionated gain of marked area in c and g. (**l**) the white light vision. Part 2. Fluorescence staining of live cells observed by confocal microscopy; (**m**) staining with rabbit anti-eEF1a MAb, left, the trophozoite shows no signal; (**n**) staining with monoclonal antibody EH3015 for Igl, left, the membrane of trophozoite (live) is intact and the other (dead) is discontinuous; (**o**) merge of m and n; (**p**) the white light vision. Bar indicates 10 μm.

A Nikon structured illumination super-resolution microscope (N-SIM) system was used to exam the co-localization of EF1a and Igl proteins (Figure 3). Images show Igl localization on the plasma membrane of all trophozoites (Figure 3b,f). Confocal microscopy indicated that Igl were on the plasma membrane, in the cytoplasm, and on intracellular vacuoles (Figure 3j). In addition, EF1a was localized in the cytoplasm (Figure 3a,e,i). Interestingly, EF1a was concentrated inside the membrane in the ectoplasmic region and had high-concentration convergence zones in the pseudopodia. Live trophozoite confocal microscopy confirmed that EF1a distributed under the membrane surface, since green fluorescence was not observed on live trophozoites (Figure 3m, left).

### 2.4. Co-Localization of EF1a and Actin on Trophozoites

**Figure 4:**
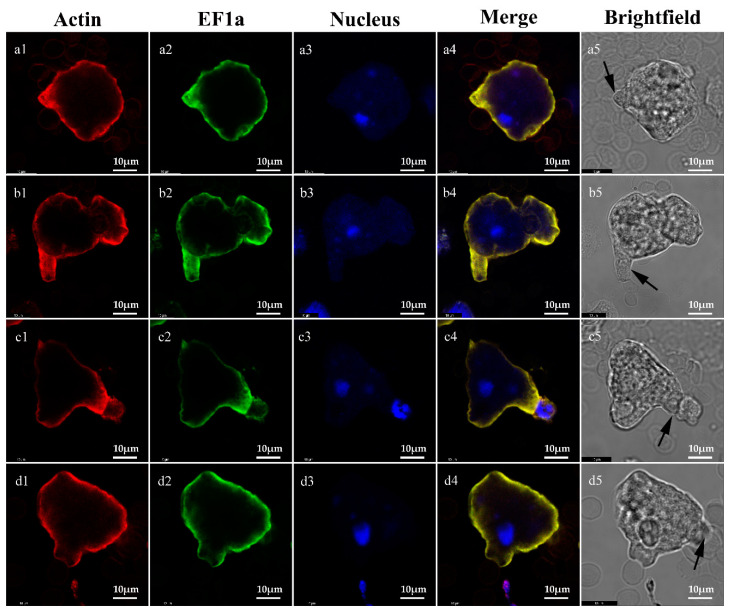
Localizations of EF1a and actin in trophozoites. EF1a was stained by indirect immunofluorescence with goat anti-mouse IgG (H + L) Cross-Adsorbed Secondary Antibody (Alexa Fluor 488); actin was stained with ActinRed™ 555 ReadyProbes™ Reagent (Rhodamine phalloidin); nuclei were stained with DAPI. (**a1**–**a5**) Co-localization in the pseudopodia; (**b1**–**b5**) co-localization at site of adhesion with two erythrocytes; (**c1**–**c5**) co-localization at site of adhesion with a monocyte; (**d1**–**d5**) co-localization at site of adhesion with erythrocytes (right). Bar indicates 10 μm.

A co-location relationship between EF1a and actin was seen with confocal microscopy in trophozoites of *E. histolytica* SAW755CR (Figure 4, Merge). EF1a is a multifunctional protein and appears to be widely distributed near the inner cell membrane (Figure 4, EF1a). Bright views indicate that co-localization occurs at sites of pseudopodia or adhesion. EF1a might act on actin in the trophozoites and participate in the regulation of actin or cytoskeleton formation.

### 2.5. Increased Expression of EF1a and Igl Genes in Trophozoites after the Co-Incubation with CHO Cells

**Figure 5:**
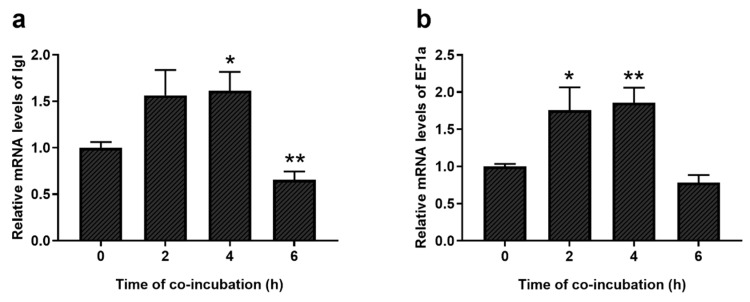
mRNA expression levels of Igl (**a**) and EF1a (**b**) of E. histolytica after the co-incubation with CHO cells for different time, as compared with control (0 h). ** *p* < 0.01, * *p* < 0.05 (Student’s *t*-test). Data are from three independent trials (mean ± SD).

Expression of EF1a and Igl genes in trophozoites after the co-incubation with CHO cells was confirmed with a real-time quantitative polymerase chain reaction (Real-time PCR) (Figure 5). Trophozoites after 2 and 4 h incubation showed a higher EF1a and Igl expression compared to controls. Further, changes in EF1a gene expression were positively correlated with changes in Igl expression, suggesting a regulatory linkage. Expression of EF1a and Igl genes displayed synchronous reduction after 6 h incubation.

### 2.6. Erythrophagocytosis Assay

**Figure 6:**
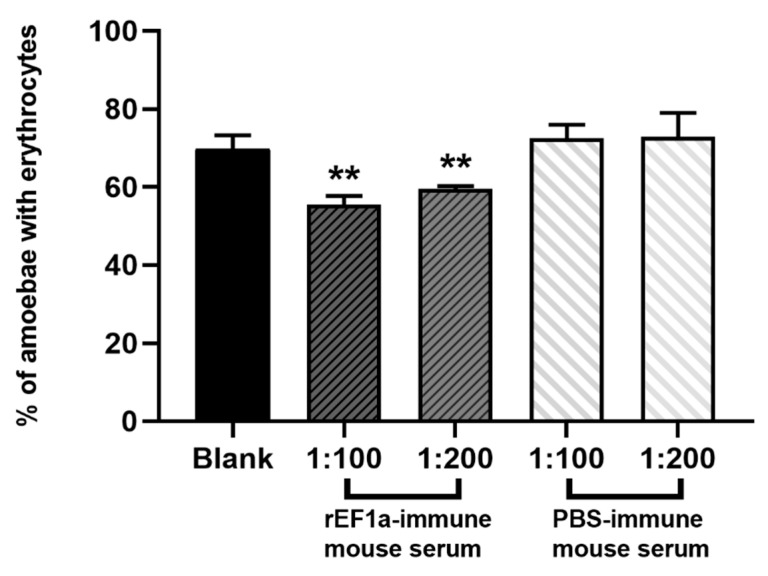
Effects of polyclonal antibodies against rEF1a on erythrophagocytosis by *E. histolytica*. Percentage of amoebae with erythrocytes based on examine of 300 amoebae (mean ± SD): group Blank, treated only with incomplete medium (69.69 ± 3.60); group treated with rEF1a-immune mouse serum (polyclonal antibodies), 1:100 (55.50 ± 2.20 **); group treated with rEF1a-immune mouse serum (polyclonal antibodies), 1:200 (59.43 ± 0.82 **); group treated with PBS-immune mouse serum, 1:100 (72.51 ± 3.44); group treated with PBS-immune mouse serum, 1:200 (72.87 ± 6.15). ** As compared with control (group treated with PBS-immune mouse serum 1:100). *p* < 0.01 (Student’s *t*-test). Data are from three independent trials.

Erythrophagocytosis assay showed a significant inhibitory effect of polyclonal antibodies against rEF1a (*p* < 0.01) (Figure 6). In contrast, sera from PBS-immune mice demonstrated no inhibitory effect. The groups treated with PBS-immune mouse serum (1:100 and 1:200) showed no statistic differences with group treated only with incomplete medium. Polyclonal antibodies against rEF1a displayed an inhibitory effect on erythrophagocytosis by *E. histolytica* trophozoites. The percentage of amoebae with erythrocytes in groups treated with polyclonal antibodies (1:100 and 1:200) were significantly reduced in a concentration-dependent manner. Experiments were performed three times to get enough data.

## 3. Discussion

Our results are consistent with collaboration between EF1a and Igl. Research on EF1a in *E. histolytica* is scarce. One report provides the use of *E. histolytica* EF1a in analyzing parasite taxonomic relationships [19], and a second provides the deduced amino acid sequence and major functional domains of the factor [18]. Our knowledge of the specific roles of EF1a in *E. histolytica* is limited, but its function in other eukaryotes may provide clues. Many studies demonstrate the multifunctional nature of EF1a protein in cellular activities [7,8,9,20], such as apoptosis, signal transduction, tumorigenesis, and cytoskeletal regulation, in addition to peptide chain extension. Pseudopodium movement, exocytosis, and endocytosis always accompany amoeba invasion, and cytoskeletal regulation by EF1a in trophozoites may be useful direction for subsequent study. Results indicated the Igl were detected on the plasma membrane, in the cytoplasm, and on intracellular vacuoles (Figure 3). Additionally, it has been confirmed that phagosomes of *E. histolytica* contain Igl and the quantity of Igl varies during the maturation of the phagosome [21]. In addition, EF1a was detected in the cytoplasm and concentrated in the pseudopodia (Figure 3). We assume that the Igl must undergo from cytoplasm to plasma membrane during the process of maturation and transport. During this routine biologically procedure, both of Igl and EF1a were detected specifically binding, suggesting that they are biologically relevant to molecular structures and transport processes. Data of real-time PCR demonstrate a consistent parallel expression of EF1a and Igl during the first four hours of incubation with CHO cells, and the erythrophagocytosis assay suggests that that EF1a have a role phagocytosis.

Interactions of EF1a and actin cytoskeleton were reported in *Dictyostelium amoebae* (slime mold) [22], where EF1a bound reversibly to cytoskeleton filaments upon stimulation. In mammalian cells, EF1a is also shown to regulate cell motility by assembling actin filaments [23,24] and regulating the activity of phosphatidylinositol 4-kinase [25]. This enzyme catalyzes the first step in the biosynthesis of phosphatidylinositol 4-phosphate (PI-4-P) and phospholipid phosphatidylinositol-4,5-bisphosphate (PI-4,5-P2). PI-4,5-P2 is a second messenger that regulates adhesion energy between the cytoskeleton and the plasma membrane [26]. The regulation of this process is fundamentally important for cell shape and a variety of cell functions. More remarkable, cytoskeletal–membrane interactions are the prime elements in formation and retraction of filopodia, lamellipodia, neurites, and other membrane processes in response to chemoattractants and other stimuli. In this study, EF1a and actin concentrate at adhesion sites or lamellipodia. Thus, EF1a might have a similar function in the regulation of the actin cytoskeleton of *E. histolytica*. Regulation of cytoskeletal functions might explain trophozoite activity after adhesion to host cells, including cytophagocytosis and the release of ameba perforin and cysteine proteinase.

In addition, EF1a may act directly on host cells. *Cryptosporidium parvum* discharges its elongation factor 1a (CpEF1a) from invading sporozoites into host cells, resulting in the formation of electron dense bands at the base of infection sites [15,16]. EF1a is also found in extracellular vehicles (EVs) of *Plasmodium berghei*. EF1a in combination with another EVs factor, termed histamine releasing factor (HRF), inhibits antigen-specific T cell responses through interference with key phosphorylation pathways associated with T cell receptor (TCR) signaling [12]. This action facilitates parasite escape from host immune responses. Finally, EF1a from *Leishmania donovani* contains a novel Src homology 2 domain with tyrosine phosphatase-1 (SHP-1) binding and activating protein both in vitro and in vivo and then limited the phagocytic function of macrophages by preventing the induction of iNOS in response to treatment with IFN-γ [27]. Immune escape also exists for amoeba pathogenicity. However, whether EF1a is involved with host cells is not known. More research is needed to explore possible mechanisms involved.

Current studies indicate that EF1a is an important drug target in eukaryotic cells. Many studies focus on the traditional role of EF1a in peptide chain extension [28,29], but some studies show that blocking the interaction between EF1a and actin can inhibit tumor migration [30]. EF1a is also considered a useful target for the study of parasite infection. Related studies in *L. mexicana* [11], *Giardia intestinalis* [13], *Toxoplasma gondii* [14], and *Trypanosoma cruzi* [17] have produced promising therapeutic and protective results. Thus, exploration of the role of EF1a in *E. histolytica* pathogenicity might also provide a theoretical foundation for drugs that target this protein. *E. histolytica* EF1a displays a hairpin structure absence from other EF1a sequences. The hairpin is associated with a 12 amino acid deletion compared with human EF1a [10,31]. Such structural specificity suggests a drug target to treat amoebiasis without affecting EF1a function in human cells. Small molecule drugs with such activity have been reported in *L. donovani* [10].

Previous and present indicate that elongation factor 1a is a synergetic partner of the intermediate subunit of Gal/GalNAc lectin in promoting phagocytosis by *E. histolytica* and might have a similar function in the regulation of the actin cytoskeleton. The specific mechanism of the synergistic effect is still unclear, but linkages among EF1a, Igl, and actin are worthy of further study.

## 4. Methods and Materials

### 4.1. E. histolytica Cell Culture

*E. histolytica* trophozoites of strain HM-1:IMSS and SAW755CR were cultivated axenically at 36.5 °C in YIMDHA-S medium [32] containing penicillin (100 U/mL), streptomycin sulfate (100 μg/mL), and 15% (*v*/*v*) heat-inactivated adult bovine serum. Trophozoites were grown for 72 h (log phase) for use in all experiments. Chinese hamster ovary cells (CHO cells) were cultured in F-12 medium with 10% fetal bovine serum (FBS), penicillin (100 U/mL), and streptomycin (100 μg/mL).

### 4.2. Pull-Down Assay and Protein Mass Spectrum

Purified Igl-C proteins (fusion protein with his6) and HisPur Cobalt Resin were incubated for at least 30 min with a gentle rocking motion on a rotating platform. All processes followed Pierce^TM^ Pull-Down PolyHis Protein: Protein Interaction Kit (Thermo Fisher, Waltham, MA, USA) instructions. One ×10^7^ HM1-1:IMSS trophozoites were harvested and thoroughly mixed with 2.5 mL of Pierce Lysis Buffer per gram wet weight of cells. After a 30 min incubation on ice, trophozoite lysates were centrifuged at 12,000× *g* for 5 min to acquire clarified supernatants. Immobilized polyhistidine-tagged bait protein (Igl-C) and prey protein (trophozoites lysis supernatant) were incubated at 4 °C for at least 1 h with gentle rocking on a rotating platform. “Prey flow-through” was collected and placed on ice after centrifugation at 1250× *g* for 30 s. The spin column was washed five times to elute the immune complex. Samples were boiled for 5 min at 100 °C. After centrifugation, proteins were separated by 12% sodium dodecyl sulfate-polyacrylamide gel electrophoresis (SDS-PAGE) and stained with coomassie brilliant blue for 30 min. The colloidal blocks were cut out and stored at 4 °C for determination of protein mass spectra. Proteomics determination was performed by Bangfei Bioscience (Beijing, China).

### 4.3. Cloning of E. histolytica EF1a Gene and Expression of Recombinant Protein

The recombinant Igl-C proteins were prepared as previously described [33]. The DNA fragment coding for full-length EF1a was obtained by PCR amplification. Oligonucleotide primers (EF1a-S 5′-3′: CCGGATCCATGCCAAAGGAAAAGACTCATATT; EF1a-AS 5′-3′: CCAAGCTTTTATTTCTTCTTTCCAGCAGCTGA) were synthesized by Invitrogen. Thirty cycles of PCR were performed as follows: denaturation at 94 °C for 15 s (195 s in cycle 1), annealing at 55 °C for 30 s, and polymerization at 72 °C for 120 s (420 s in cycle 30). Amplified DNA fragments were digested with *Bamh*I and *Hind*III, purified, and ligated with the pQE-30 vector (QIAGEN, Hilden, Germany). Recombinant plasmids were introduced into competent *Escherichia coli* JM109 cells. Clones containing the correct sequence of inserts were selected. Recombinant plasmids were transformed into *E. coli* M15 cells. A single clone was selected and cultured in 800 mL of Luria-Bertani (LB) medium containing ampicillin. When an optical density at 600 nm (OD600) of 0.6 was achieved, bacteria were induced with isopropyl-β-d-thiogalactopyranoside at a final concentration of 1 mM and incubation continued at 30 °C for 6 h. Bacteria were then centrifuged and suspended in 15 mL of binding buffer (20 mM Tris-HCl [pH 7.9], 0.5M NaCl, 5 mM Imidazole) with phenylmethanesulfonyl fluoride (PMSF, final concentration of 1 mM). After sonication at 4 °C, lysates were centrifuged, and supernatants containing the recombinant EF1a protein (rEF1a) were further purified with a Ni-NTA His Bind Resins kit (Novagen, Madison, WI, USA). The purity of the recombinant protein was analyzed by SDS-PAGE under reducing conditions [34].

### 4.4. Biolayer Interferometry Binding Assays

Binding kinetics of rEF1a with Igl-C protein or an unrelated protein (bovine serum albumin, BSA) were analyzed by biolayer interferometry using an Octet-Red96 device (Pall ForteBio, Menlo Park, CA, USA). Purified Igl-C and BSA in 30 μg/mL solutions of buffered sodium acetate (pH 4.0) and sodium acetate (pH 5.0), respectively, were immobilized onto activated AR2G biosensors until saturation. Twofold serial dilutions of rEF1a protein were prepared from 4000 nM to 250 nM. AR2G biosensors were then incubated with the serial dilutions in running buffer at pH 7.4. Assays were performed in the following steps at 30 °C: (1) equilibration with water for 60 s; (2) activation of AR2G biosensors by 20 mM 1-ethyl- 3-(3-dimethylaminopropyl) carbodiimide hydrochloride and 10 mM N-hydroxysuccinimide for 300s; (3) immobilization of protein onto sensors for 300 s; (4) quenching with 1Methanolamine (pH 8.5) for 300 s; (5) baseline step in 1× Kinetics buffer for 120 s; (6) association of rEF1a for measurement of K_on_ for 600 s; (7) dissociation of rEF1a for measurement of K_off_ (700 s). Association and dissociation were measured and fitted based on a 1:1 binding kinetic model with ForteBio Data Analysis software.

### 4.5. Monoclonal Antibody Evaluation with the Western Blotting

Anti-Igl EH3015 [3] is a mouse MAb, and a rabbit anti-eEF1a MAb (catalog no. ab157455; Abcam, Cambridge, UK) was used to localize the *E. histolytica* EF1a in trophozoites. EF1a is a conserved protein; still, Western immunoblotting is necessary. *E. histolytica* HM1-1:IMSS trophozoites of 5 × 10^6^ cells/mL were solubilized with an equal volume of sample buffer containing 2 mM phenylmethylsulfonyl fluoride, 2 mM N-α-ρ-tosyl-L-lysine chloromethyl ketone, 2 mM ρ-hydroxymercuriphenylsulfonic acid, and 4 μM leupeptin for 5 min at 95 °C [35]. The supernatant was subjected to Glycine–SDS-PAGE in 10% polyacrylamide gel. Precision Plus ProteinTM Standards (Bio-Rad, Hercules, CA, USA) were used as molecular mass markers. Western immunoblotting analysis was performed as previously described [36]. Rabbit anti-eEF1a Mab (catalog no. ab157455; Abcam, Cambridge, UK) was used as the primary antibody, and horseradish peroxidase-labeled goat anti-rabbit IgG was used as the second antibody. Development was performed by with an enhanced HRP-DAB Chromogenic Substrate Kit (TIANGEN, Beijing, China).

### 4.6. Mouse Original Polyclonal Antibodies against rEF1a

Mouse original polyclonal antibodies against rEF1a were prepared after immunization with target proteins described in a previous study [37]. The specificity of polyclonal antibodies examined by Western blotting following the process described above, using mouse anti-rEF1a polyclonal antibodies (1:4000) as the primary and goat anti-mouse IgG as the secondary antibody.

### 4.7. Fluorescence Imaging of Trophozoites

After chilling on ice for 3 min, trophozoites of strain HM-1:IMSS were centrifuged at 500× *g* for 3 min. Pellets were resuspended in phosphate-buffered saline (PBS) containing 2% (*w*/*v*) glucose. Trophozoites were incubated in a water bath at 37 °C for 3 min then fixed in preheated 4% paraformaldehyde (PFA) solution and permeabilized in 0.1% Triton X-100 for 5 min. Permeabilized cells were incubated in blocking agent (3% bovine serum albumin in PBS) for 1 h at room temperature. Subsequently, trophozoites were incubated with a primary antibody, either rabbit anti-eEF1a MAb (catalog no. ab157455; Abcam, Cambridge, UK) (2.6 μg/mL) or anti-Igl EH3015 (50 μg/mL) [3] in blocking agent overnight at 4 °C. Cells were then incubated with 1:200 dilutions of the appropriate secondary antibody in blocking agent: goat anti-rabbit IgG (H + L) Cross-Adsorbed Secondary Antibody (Alexa Fluor 488, Catalog no.A-11008, Invitrogen, Carlsbad, CA, USA) or goat anti-human IgG (H + L) Cross-Adsorbed Secondary Antibody (Alexa Fluor 594, Catalog no.A-11014, Invitrogen). The trophozoites were then resuspended in 10% glycerin–PBS. An N-SIM microscopy system (Nikon, Tokyo, Japan) laser scanning confocal microscope (Leica, Wetzlar, Germany) was used to visualize and compile final images with laser excitation at 488 nm for EF1a or 561 nm for Igl. To determine whether EF1a was also distributed on the membrane surface, live trophozoite staining was also performed without fixing or permeabilization and visualized with a laser scanning confocal microscope (Leica, Wetzlar, Germany).

The SAW755CR strain was used to explore the location of EF1a and actin for its active motility. Trophozoites were centrifuged at 500× *g* for 3 min. Type O human red blood cells were prepared and washed twice with PBS. Pellets were resuspended in YIMDHA-S medium without serum at a concentration of 10^6^ /mL (trophozoites) and 10^8^ /mL (erythrocytes). 10^5^ trophozoites and 10^7^ red blood cells were mixed and incubated for 30 s at 37 °C and then fixed in preheated 4% PFA for 30 min at room temperature. Actin was dyed with ActinRed™ 555 ReadyProbes™ Reagent (Rhodamine phalloidin) after treatment in 0.5% Triton X-100 for 10 min. The trophozoites were incubated in a blocking agent (3% bovine serum albumin in PBS) for 30 min at room temperature. Indirect immunofluorescence used mouse anti-rEF1a polyclonal antibodies (1:100) as the primary antibody and goat anti-mouse IgG (H + L) Cross-Adsorbed Secondary Antibody (Alexa Fluor 488, Catalog no. A-11029, Invitrogen, Carlsbad, CA, USA)), as described above. Results were imaged with a laser scanning confocal microscope (Leica, Wetzlar, Germany)

### 4.8. Co-Incubation of Trophozoites and CHO Cells

Changes of gene expression in HM1-1:IMSS trophozoites during invasion were examined in CHO cell in culture. CHO cells were released with 0.25% trypsin digestion and subcultured into 25 cm^2^ cell culture flasks. *E. histolytica* trophozoites were incubated on ice for 3 min. After centrifugation at 500× *g* for 3min, trophozoites were resuspended in YIMDHA-S medium containing 15% (*v*/*v*) heat-inactivated adult bovine serum. CHO cells were then washed once with YIMDHA-S medium before adding *E. histolytica* suspension [38]. About 5 × 10^6^ trophozoites were transferred to each 25 cm^2^ flask, and warm YIMDHA-S medium was added to flask to bring the total volume to 40 mL. Co-incubation of trophozoites with CHO cells continued in an anaerobic environment at 36.5 °C for up to 6 h. After chilling on ice for 3 min, trophozoites were harvested at 0, 2, 4 and 6 h after the start of incubation.

### 4.9. Gene Expression of EF1a and Igl by Real-Time PCR

Gene expression of EF1a and Igl in HM1-1:IMSS trophozoites was examined by real-time PCR using the primers listed in Table S2. Briefly, total RNA (1 μg) from trophozoites was purified with a RNeasy^®^ Plus Mini Kit (QIAGEN, Hilden, Germany). One-step reverse transcription PCR (one-step RT-PCR) was performed using a PrimeScript™ One-step RT-PCR Kit (Takara, Kyoto, Japan). cDNA was synthesized with a PrimeScript first-strand cDNA synthesis kit (Takara, Kyoto, Japan) using oligo (dT) primers. Real-time PCR was carried out in a final reaction volume of 20 μL with an ABI 7500 real-time PCR system (Applied Biosystems, Carlsbad, CA, USA). Reactions were performed in a 96-well plate with SYBR Premix Ex Taq (Takara, Kyoto, Japan) containing primers listed in Supplementary Table S2. The amplification cycling conditions were: 30 s at 95 °C and 40 cycles of 5 s at 95 °C and 35 s at 60 °C. Analysis of gene expression was conducted during the log phase of product accumulation, during which Ct values correlated linearly with relative DNA copy numbers. Each experiment was performed at least three times.

### 4.10. Effect of EF1a on the Amebic Pathogenicity with the Erythrophagocytosis Assay

Initially, 1.25 × 10^6^ HM-1:IMSS trophozoites were washed with cold PBS after a 5 min ice bath and divided into five equal groups. After centrifuging at 500× *g* for 3 min, trophozoites were resuspended in a 500 μL incomplete YIMDHA-S medium without adult bovine serum. Trophozoites in two groups were exposed to anti-rEF1a polyclonal antibodies with 5 μL (1:100) and 2.5 μL (1:200) rEF1a-immune mouse serum. Trophozoites in another two groups were exposed to 5 μL (1:100) and 2.5 μL (1:200) PBS-immune mouse serum. Trophozoites in the last group served as a blank control. All trophozoites were incubated at 37 °C for 30 min. Type O human erythrocytes were prepared and washed twice with PBS. Erythrophagocytosis assay was performed by mixing pretreated trophozoites with erythrocytes at a ratio of 1:100, followed by incubation at 37 °C for 5 min. Distilled water was added immediately to lyse free and adherent erythrocytes. Trophozoites were then fixed in 2.5% glutaraldehyde for 30 min at room temperature. Engulfed erythrocytes were stained with a DAB solution containing 0.2% H_2_O_2_. Numbers of trophozoites with ingested erythrocytes was determined by examining 300 amoebae.

### 4.11. Ethical Statement

To prepare mouse original polyclonal antibodies, animal experiments were performed in strict accordance with the Regulations for the Administration of Affairs Concerning Experimental Animals (1988.11.1) and were approved by the Institutional Animal Care and Use Committee (IACUC) of our institutions (Permit Numbers 20160225-097). Collection of type O human peripheral blood was approved by The Institutional Ethics Committees of our institutions (Permit Numbers 2018-Y022).

## Supplementary Materials

The following are available online at https://www.mdpi.com/2076-0817/9/9/702/s1, Table S1: Protein mass spectrum, Table S2: Sequences of primers used in real-time PCR.
