# Supplementary material for: Evaluation on Elongation Factor 1 Alpha of Entamoeba histolytica Interaction with the Intermediate Subunit of the Gal/GalNAc Lectin and Actin in Phagocytosis
Source: Pathogens. 2020 Aug 27;9(9):702. doi: 10.3390/pathogens9090702 (PMC7558290; doi:10.3390/pathogens9090702)
Supplement: Supplementary file 1 [file pathogens-09-00702-s001.zip › pathogens-901576-supplementary(Round4)/Table S1.docx]

**Table S1.** Protein mass spectrum

|  | Reference | PepCount | UniquePepCount | CoverPercent | MW | PI |
| --- | --- | --- | --- | --- | --- | --- |
| 1 | sp\|O76156\|SAT_ENTHI Sulfate adenylyltransferase OS=Entamoeba histolytica PE=3 SV=1 | 89 | 40 | 70.21% | 47927.23 | 9.17 |
| 2 | tr\|S0B1J5\|S0B1J5_ENTHI Sulfate adenylyltransferase, putative OS=Entamoeba histolytica PE=2 SV=1 | 77 | 40 | 70.21% | 47929.2 | 9.17 |
| 3 | **tr\|A0A060N2B5\|A0A060N2B5_ENTHI Elongation factor 1-alpha OS=Entamoeba histolytica PE=2 SV=1** | 394 | 39 | 74.66% | 48493.35 | 8.98 |
| 4 | **tr\|S0AV64\|S0AV64_ENTHI Elongation factor 1-alpha OS=Entamoeba histolytica PE=2 SV=1** | 393 | 39 | 78.96% | 48431.39 | 9.13 |
| 5 | **tr\|S0AWG4\|S0AWG4_ENTHI Elongation factor 1-alpha OS=Entamoeba histolytica PE=2 SV=1** | 393 | 39 | 78.96% | 48389.27 | 9.08 |
| 6 | **tr\|S0B0R7\|S0B0R7_ENTHI Elongation factor 1-alpha OS=Entamoeba histolytica PE=2 SV=1** | 385 | 38 | 78.96% | 48405.26 | 9.08 |
| 7 | **tr\|S0AYN6\|S0AYN6_ENTHI Elongation factor 1-alpha OS=Entamoeba histolytica PE=2 SV=1** | 363 | 33 | 65.61% | 48445.31 | 9.05 |
| 8 | tr\|S0AVH8\|S0AVH8_ENTHI Ribosomal protein S4, putative OS=Entamoeba histolytica PE=2 SV=1 | 30 | 19 | 46.01% | 36756.2 | 10.33 |
