# Supplementary material for: Evaluation on Elongation Factor 1 Alpha of Entamoeba histolytica Interaction with the Intermediate Subunit of the Gal/GalNAc Lectin and Actin in Phagocytosis
Source: Pathogens. 2020 Aug 27;9(9):702. doi: 10.3390/pathogens9090702 (PMC7558290; doi:10.3390/pathogens9090702)
Supplement: Supplementary file 1 [file pathogens-09-00702-s001.zip › pathogens-901576-supplementary(Round4)/Table S2(Round4).docx]

**Table** **S2** Sequences of primers used in real-time PCR

| Primer Name | Primer sequence (5′–3′) |
| --- | --- |
| Eh18s-specific-F | AGGTAGTGACGACACATAAC |
| Eh18s-specific-R | CTTACATAAAGTCTTCAAAAT |
| Eh-EF1a-F | GCACTTGATTCAGTCACACCAC |
| Eh- EF1a-R | CAGATGAAACTCCTGATGGTGC |
| Igl-3'-F[39] | TGAAGGCACTTCTACAGAAGATAATAAAAT |
| Igl-3'-R | TATGTCTTGAACATGGAATACATGATC |

[39] Linford, A.S.; Moreno, H.; Good, K.R.; Zhang, H.; Singh, U.; Petri, W.A. Short hairpin RNA-mediated knockdown of protein expression in Entamoeba histolytica. BMC Microbiol. 2009, 9, 38.
